# Targeting Insulin and Insulin-Like Growth Factor Pathways in Epithelial Ovarian Cancer

**DOI:** 10.1155/2010/257058

**Published:** 2010-01-05

**Authors:** Marie-Claude Beauchamp, Amber Yasmeen, Ariane Knafo, Walter H. Gotlieb

**Affiliations:** ^1^Division of Gynecologic Oncology, Jewish General Hospital, McGill University, Montreal, QC, Canada H3T 1E2; ^2^Segal Cancer Center, Lady Davis Institute of Medical Research, McGill University, Montreal, QC, Canada H3T 1E2

## Abstract

Ovarian cancer is the most lethal of all gynecological malignancies, due in part to the diagnosis at an advanced stage caused by the lack of specific signs and symptoms and the absence of reliable tests for screening and early detection. Most patients will respond initially to treatment but about 70% of them will suffer a recurrence. Therefore, new therapeutic modalities are urgently needed to overcome chemoresistance observed in ovarian cancer patients. Evidence accumulates suggesting that the insulin/insulin growth factor (IGF) pathways could act as a good therapeutic target in several cancers, including ovarian cancer. In this paper, we will focus on the role of insulin/IGF in ovarian cancer tumorigenesis and treatment.

## 1. Introduction

Ovarian cancer is the leading cause of death among all gynecological cancers in western countries. When compared to other gynecological cancers, the fatality rate of ovarian cancer surpasses that of cervical and endometrial cancers put together [1]. This high death rate is due to the diagnosis at an advanced stage in most patients caused by the relative lack of specific signs and symptoms of the disease and the lack of reliable tests for early detection. It is estimated that this year in North America, 24 150 women will be newly diagnosed with ovarian cancer and that 17 220 women will die of the disease [2]. Epithelial ovarian cancer (EOC) constitutes 90% of ovarian malignancies and is classified into distinct histologic categories including serous, mucinous, endometrioid, clear cell, transitional, mixed, and undifferentiated subtypes [3]. Nowadays, data suggest that the cell of origin for an important proportion of high-grade pelvic serous carcinomas, including the ovary, is derived from the distal fallopian tube [2]. 

Although most patients with EOC experience a reasonable initial clinical response to debulking surgery and chemotherapy, the majority of these patients will not be cured. Approximately 70% will experience a recurrence and this chemoresistance is responsible for the majority of ovarian cancer-related deaths [4]. Presently, there are no available treatments capable of curing recurrent ovarian carcinomas due to their rapid evolution into a chemoresistant disease. It has therefore become essential to introduce new therapeutic modalities that will change response to treatment into cure and salvage these patients. Over the last decade, accumulating data suggest that the insulin/IGF pathway might be one such good therapeutic target in cancers, including ovarian cancer. In this paper, we intend to review the role of insulin/IGF pathway in ovarian cancer and the various strategies to target it.

## 2. Physiological Roles of Insulin and Insulin-Like Growth Factor

Insulin and Insulin-like growth factor (IGF) signaling regulates cellular growth, proliferation, metabolism, and survival. Insulin was discovered in 1922 and is a crucial regulator of metabolic pathways. It is under the tight control of blood glucose levels and is excreted by the pancreas solely in periods of rising blood glucose levels [5]. When released by the beta-cells of the pancreas, insulin binds to receptors on the surface of most cells. Hepatocytes, adipocytes, and muscle cells are classic insulin responsive cells and express high levels of insulin receptors. Insulin is primarily involved in regulating metabolism but was also shown to have a mitogenic effect [6]. On the other hand, IGF signaling plays a fundamental role in regulating embryonic growth and regulates specific differentiation in most adult tissues [7]. IGF is a major downstream target of growth hormone (GH) and is essential for regulating growth and body size both in the prenatal and postnatal stage [8]. The insulin and IGF-I receptors, though separate gene products, are structurally very similar. In addition, insulin and IGF-I are closely related peptides. Amino acid similarities range between 40 and 85% in different domains with the highest degree of homology being found in the tyrosine kinase domain [9].

Interestingly, the expression, signaling mechanisms, and roles of members of the insulin/IGF family such as ligands, receptors, binding proteins, and binding protein proteases and their inhibitors have been elucidated in ovarian follicle function in humans and other species. In vitro studies and genetic approaches using mouse knockout models for IGF family members have revealed that IGFs are key intraovarian regulators of follicular growth, selection, atresia, cellular differentiation, steroidogenesis, oocyte maturation, and cumulus expansion [10]. Some of these actions are synergistic with gonadotropins, although most are not sustainable with IGFs alone and require gonadotropin actions. In fact, IGFs are designated as copartners of gonadotropins. Moreover, recent studies demonstrate that endocrine-disrupting chemicals can compromise IGF activity and signaling in the ovarian follicle, affecting follicular development, steroidogenesis, and oocyte quality. The successful development of a healthy oocyte and appropriate granulosa and theca cell steroidogenesis on a cyclic basis are contingent on multiple factors, including a properly functioning of intraovarian IGF system [11]. Disruption of even one component of this system can lead to abnormal follicular development and function. Interaction of the IGF system with other growth factor systems and ovarian peptides during follicular development is still in early investigative stages.

## 3. Insulin and IGFs Structure and Signaling

### 3.1. Insulin and IGF Ligands

Insulin/IGF signaling system is comprised of three ligands, IGF-I, IGF-II, and insulin itself. These ligands interact with at least four receptors: the type I IGF receptor (IGF-IR), the type II IGF receptor (IGF-IIR), the insulin receptor (IR), hybrid receptors of IGF, and insulin [12]. The circulating and biologically active form of insulin ligands is a monomer consisting of two chains, an A chain of 21 amino acids and a B chain of 30 amino acids linked by two disulfide bridges [13]. On the other hand, IGFs are small, single-chain polypeptide ligands (7-8 kD) that are derived from prepropeptides in a similar way to insulin, but contain the C-peptide bridge between B and A chains that is normally cleaved in insulin [14]. The mature IGF-I and IGF-II peptides consist of B and A domains that are homologous to B and A chain of insulin.

### 3.2. Insulin and IGFs Receptors and Signaling

Insulin action is mediated through its receptor. The IR is a heterotetrameric protein consisting of two extracellular *α*-subunits and two transmembrane *β*-subunits. The binding of ligand to the *α*-subunits of IR stimulates the intrinsic tyrosine kinase activity of the *β*-subunits of the receptor [15]. The ability of the receptor to autophosphorylate and phosphorylate intracellular substrates is essential for the mediation of the complex cellular responses to insulin. The activated IR tyrosine kinase phosphorylates several immediate substrates including insulin receptor substrate proteins (IRS1-4), DOK4, DOK5, SHC, Gab1, Cbl, APS, and signal regulatory protein family. These adaptor proteins provide an interface between the activated receptors and the downstream-located effector molecules. Insulin activates the mitogenic (via MAP kinases and Erk1/2) and metabolic branches of insulin signaling, the latter involving PI3 kinase, PKB/Akt, mTORC1, p70S6 kinase, as well as PLC*γ* [16–18]. There are two isoforms of IR that are involved in different cellular functions. These two isoforms of IR are generated by alternative splicing of exon 11, giving rise to the B-isoform (IR-B) and A-isoform (IR-A) [19]. They are expressed in a developmentally specific manner, with high expression of IR-A in fetal tissues and IR-B in adult tissues. Moreover, IGF-II binds IR-A with high affinity whereas IGF-I does not [20, 21]. 

The IGF-I and IGF-II ligands interact with an array of cell receptors that may be present singly or in various combinations on target cells. IGF-I has a twofold higher affinity for the IGF-IR than for the IR, most of the effects of IGF-I result from activation of the IGF-IR. IGF-I and IGF-II interact with the IGF-IR, a transmembrane tyrosine kinase that is structurally and functionally related to the IR [21, 22]. Homology between IR and IGF-IR ranges 45–65% and 60–85% for the ligand binding, tyrosine kinase, and substrate recruitment domains, respectively [23]. Ligand binding of IGF-I or IGF-II to IGF-IR results in a conformational change leading to transphosphorylation of one *β*-subunit by the other. Activated IGF-IR recruits and phosphorylates adaptor proteins belonging to the insulin receptor substrate (IRS) family or SHC. The phosphorylated adaptor proteins then serve as docking sites for other signaling molecules, resulting in the activation of the downstream pathways. The IGF-1R plays a central role in integrating signals of nutrition and stress into energy shifts from energy expensive anabolic processes such as growth and reproduction [12, 24].

IGF-IIR is a multifunctional receptor that lacks an intracellular signaling domain. It is known as the cation-independent mannose-6-phosphatase receptor that binds to a diverse group of mannose-6-phosphatase tagged proteins for endosomal trafficking and degradation by the lysosome. The IGF-IIR or the cation-independent mannose-6-phosphate receptor binds IGF-II and causes internalization and subsequent clearance by the lysosome. IGF-IIR is involved in the regulation of the extracellular concentration of IGF-II [25]. 

Furthermore, many cells and tissues have hybrid receptors assembled with one chain of the IGF-IR and one of the IR. IGF-IR/IR-B hybrids have higher affinity for IGF-I whereas IGF-IR/IR-A hybrids have equal affinity for IGF-II and insulin. Insulin binding to hybrid receptors initiates similar cellular responses as when binding to IR or IGF-IR. In both cases, ligand binding to their receptors will stimulate the activity of their intrinsic tyrosine kinase [26, 27]. However, the exact role of hybrid receptors in signaling needs further investigation.

### 3.3. Insulin-Like Growth Factor Binding Proteins (IGFBPs)

The IGFs action is under the control of six binding proteins. IGFBPs are a family of secreted proteins that bind IGFs with equal of greater affinity than to IGF-IR. Six designated IGFBPs (1–6) have been isolated and characterized so far in human and in a variety of vertebrate species. These IGFBPs, with apparent molecular mass of 24–45 kDa, share a common domain organization. All of them have a highly conserved N-terminal domain, a conserved C-terminal domain, and a variable central linker domain. Most IGFBPs function as carrier proteins for circulating IGFs and regulate IGF turnover, transport, and tissue distribution, thus determining the physiological concentration of IGFs. Another important role of IGFBPs may be to help in the storage of IGFs in the extracellular matrices of certain tissues [28].

IGFBPs are produced by a variety of biological tissues and are thus found in various biological fluids. Although all six known IGFBPs belong to the same gene family, several features distinguish IGFBPs from each other. IGFBP-1, IGFBP-2, IGFBP-4, and IGFBP-6 inhibit IGFs actions by preventing their binding to IGF receptors. In the circulation, IGF-I and IGF-II are mainly bound to IGFBP-3, which is the most abundant IGFBP in serum. Moreover, IGFBP-3 was found not only to regulate the mitogenic actions of IGFs but also to inhibit their antiapoptotic effect. Intriguingly, IGFBP-3 has been localized in the nucleus, implying a more direct transcriptional regulatory role, but the way extracellular IGFBP-3 enters the cell remains largely unknown. IGFBPs bind to IGF-I and IGF-II with the same affinity as the latter do with IGF-IR [29, 30]. Under different physiological conditions, the IGFBPs can either increase or decrease IGF signaling, probably related to the fact that IGFBPs can prolong the half-lives of IGFs but also can compete with receptors for free IGF-I and IGF-II. However, IGFBP-1, IGFBP-3, and IGFBP-5 can also mediate their effects on the target cells by an IGF independent pathway [31]. Table 1 summarizes the physiological roles of each insulin/IGF family members.

An additional important variable is the presence of specific IGFBP proteases. IGFBPs have been reported to be proteolytically degraded by a variety of serine and matrix metalloproteases. Proteolytic activity has been described for IGFBP-2, IGFBP-3, IGFBP-4, and IGFBP-5. Since the IGFBP fragments that are generated bind IGF-I weakly or not at all, proteolysis is believed to play an important role in controlling the bioavailability of IGF-I to receptors at the cellular level. Although fragments that are generated usually have reduced affinity for the IGFs, the cleavage of IGFBP-3 generates a 30-kDa fragment with relatively intact affinity for IGF-II [32]. This raises the possibility that these proteases may function to release IGFs, making them available to bind to receptors. Overall, the bioavailability and biological activity of IGFs are modulated by these IGFBPs and their proteases. 

## 4. Insulin/IGFs in Human Cancers

IGF ligands, receptors, and IGFBPs have been shown to play a critical role in the development and progression of human cancers. Elevated plasma concentrations of IGF-I or IGFBP-3 have been linked to a high risk for several types of cancers including breast, prostate, and lung cancer [33–35]. In addition, the expression levels of the IGF-IR and IR are predictive of breast cancer outcome. Several studies have also reported that inhibition of IGF-IR reduces metastasis of various cancer cells emphasizing the importance of IGF signaling in cancer progression. IGF/IGF-IR have been studied extensively in metastatic colon, pancreatic, prostate, and breast cancer [21, 36]. In many human cancers, there is a strong association with dysregulated insulin/IGF signaling pathway that has been extensively reviewed. However, the role of insulin/IGF in ovarian cancer warrants further description.

## 5. Components of the IGF Axis Expression in Human Ovarian Cancer Risk

The first study showing the expression of IGF-I mRNA in ovarian cancer cells and tissues was published back in 1991 by Yee et al. [37]. They also reported several IGFBPs and the IGF-IR expression by ovarian cancer cells. This study suggested that all necessary components for an IGF-I-mediated autocrine loop are present in ovarian cancer cells, an observation that was also confirmed in one of our early studies using the OVCAR-3 cell line [38]. Two other groups described the expression of the IGF and insulin receptors in ovarian tumors [39, 40]. During the same period, it was reported that IGF-I levels were higher in cyst fluid from invasive malignant neoplasms compared to benign tumors [41]. Later, another group confirmed the presence of the IGF-IR expression by immunohistochemistry (IHC) in 100% of the ovarian carcinomas samples tested [42]. These initial studies opened the door to a widespread area of research in ovarian cancer, indicating an involvement of the insulin/IGF system in ovarian tumorigenesis. 

### 5.1. Tissue Expression of the Insulin/IGF System in Ovarian Cancer

A strong support for a role of IGF-I in ovarian cancer progression came from a recent study by Brokaw et al., who showed that high free IGF-I protein expression in ovarian tumor tissue was independently associated with the progression of ovarian cancer [43]. Moreover, IGF-I mRNA expression was also associated with disease progression, implying that both endocrine and paracrine/autocrine regulations of IGF-I activity are involved in ovarian cancer [43]. Similarly, microarray expression profiles from 64 EOC patients demonstrated that individual genes including IGF-I, IGF-IR, and several genes downstream of the receptor were overexpressed in tumors associated with an unfavorable prognosis [44].

Another member of the IGF family that seems to be involved in ovarian cancer is the IGF-II. It has been reported that IGF-II gene expression is increased more than 300-fold in cancer tissues compared to normal ovarian surface epithelium (NOSE) samples [45]. Interestingly, two studies showed that IGF-II is associated with disease progression, and proposed that it can be a predictor of poor survivals for patients with EOC [45, 46]. Recently, the protein expression of IGF-II mRNA-binding protein 3 (IGF2BP3, also known as IMP3) was reported to be an independent marker for reduced disease-specific survival in the rarely studied clear cell carcinoma subtype of ovarian cancer [47]. 

Finally, it was demonstrated that IGFBP-2 relative mRNA expression was 38-fold higher in ovarian cancer than in NOSE [48]. A concomitant elevation in serum IGFBP-2 was also observed in cancerous specimens, conveying the notion that IGFBP-2 might represent a novel biomarker for detection and/or monitoring of EOC [48]. In opposition to the above described studies, serum IGFBP-3 levels are decreased in patients with ovarian cancer [49] and low IGFBP-3 levels are associated with a higher risk for disease progression [50] and poor survival [51]. The studies mentioned above are detailed in Table 2.

### 5.2. Circulating Levels of the Insulin/IGF System in Ovarian Cancer

In the same order of idea, a lot of efforts were made to verify the use of certain components of the IGF system expression as predictive markers for ovarian cancer. Thus, IGFBP-2 levels were determined in the serum of EOC patients and found to positively correlate with cancer antigen 125 (CA125) [49], a widely used marker for ovarian cancer follow-up. Overall, in retrospective studies, lower IGF-I levels were found in serum of disease patients versus controls [41, 49, 52–55]. 

On the other hand, two recent prospective studies reported a higher ovarian cancer risk among women aged 55 or less at time of diagnosis when comparing the top and bottom tertile of IGF-I levels [56, 57]. However, in a recent nested case-control study using data from three prospective cohorts, namely, the Nurses' Health Study (NHS), NHSII, and the Women's Health Study (WHS), no significant positive association between IGF related proteins (IGFBP-2, IGFBP-3, and IGF-I) and ovarian cancer risk was found [58]. 

In general, studies aimed at determining an association between ovarian cancer risks and circulating IGF concentrations have been few and inconsistent [59] (Table 3). Clearly more investigative efforts are needed to confirm the role of this hormone in ovarian cancer although biological evidence suggests a mitogenic role of insulin and IGF-I in the development of this disease.

## 6. Role of IGF Family in Ovarian Carcinogenesis: Proliferation, Angiogenesis, Invasion, and Metastasis

A primary study using ovarian cancer cell lines implicated IGF-II in cell adhesion and invasion through the stimulation of the extracellular matrix glycoprotein tenascin-C [60]. Later, accumulating evidence depicted a role for IGF-I in cellular proliferation, invasion, and angiogenesis. Firstly, Shen et al. demonstrated an induction of KCl Cotransport (KCC) in response to IGF-I in OVCAR-3 cells. This KCC was necessary for IGF-I-induced cancer cell invasiveness and proliferation [61].

Next, the induction of cell invasion and proliferation by IGF-I occurred through phorpshorylation of AKT and ERK1/2 in human ovarian cancer cells HRA [62]. IGF-I also induced cyclooxygenase-2 (COX-2), a crucial player in tumor angiogenesis, partly by enhancing vascular endothelial growth factor (VEGF) production [63]. This elevation of COX-2 expression was followed by an augmentation of prostaglandins E_2_ (PGE_2_) biosynthesis and was associated with the activation of PI3K, MAPK, and PKC pathways. Finally, IGF-I and insulin stimulated the migration of SKOV-3 cells by favoring the urokinase-type plasminogen activator (uPA) over the plasminogen activator inhibitor-1 (PAI-1) through the PI3K/AKT pathway [64]. An induction of uPA is linked to a poor prognosis and correlates to a more aggressive phenotype of ovarian cancer [65–68]. As stated earlier, IGFBP-2 is overexpressed in ovarian malignant tissues and in the serum and cystic fluid of ovarian cancer patients [41, 48, 49, 69], indicating a role in the biology of ovarian cancer. Indeed, it was reported that IGFBP-2 stimulated the invasion of SKOV-3 cells using the Matrigel invasion assay, an effect reversible by an attenuation of its expression by small interference RNA (siRNA) [70]. 

On the contrary, two IGFBPs seem to have a suppressing effect on invasion, metastasis, and angiogenesis. Interestingly, it was recently shown that IGFBP-3 inhibited cell migration, invasion, and metastasis in the human ovarian endometrioid carcinoma cell line OVRW59-P4 [51], an observation that correlates with the low levels of IGFBP-3 expression in high tumor grade, advanced stage, and poor survival in endometrioid carcinoma and EOC patients [50, 51]. IGFBP-5 function in angiogenesis was also studied in a xenograft model of ovarian cancer. IGFBP-5 expression prevented tumor growth and tumor vascularity, indicating a tumor suppressor role in ovarian cancer [71].

## 7. Development of Inhibitors of the Insulin/IGF-I Pathways

**Figure 1:**
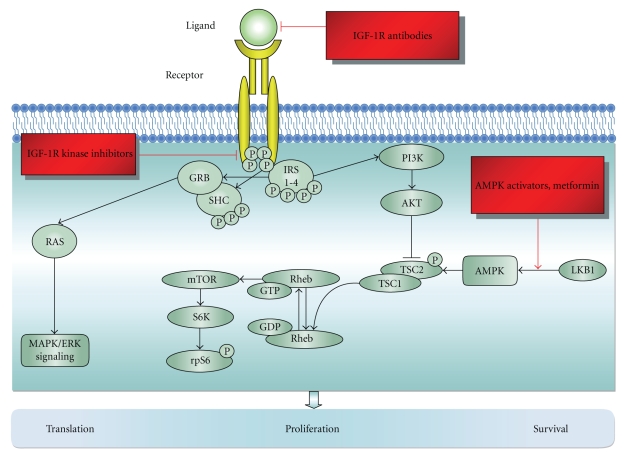
IR and IGF family signaling pathway. Upon the binding of the ligand, the activated receptor will undergo autophosphorylation and in turn will phosphorylate IRS and SHC. Activated IRS will recruit GRB to the phosphorylated form of SHC adaptor protein. The SHC-GRB complex will induce RAS and turn on the MAPK/ERK pathway, inducing cell proliferation and survival. Phospho-IRS will also stimulate the PI3 kinase to phosphorylate AKT thus initiating its downstream effectors such as mTOR, promoting translation, proliferation, and cell survival. Generally, activated AKT will have an inhibitory effect on TSC2, allowing Rheb-GDP to be converted to its GTP-bound state, thereby activating mTOR and its downstream signaling molecules to promote cellular translation. Three different potential targeted therapies are underway of investigation in ovarian cancer, including IGF-IR antibodies, IGF-IR kinase inhibitors, and AMPK activators such as metformin.

The strategies to target IGF in cancer consist of (1) reducing circulating ligand levels or bioactivity, (2) blocking receptor function using receptor-specific antibodies or small-molecule tyrosine kinase inhibitors, and (3) activating AMP-activated protein kinase (AMPK) (see Figure 1). 

### 7.1. Ligand-Targeted Approach

The first-generation strategies that included the use of somatostatin analogues to diminish circulating IGF-I levels were unsuccessful [7]. It was reported in one of the largest clinical trials that the suppression of ligand levels was not achieved using this approach [72], suggesting a failure of this particular strategy rather than an evidence of a wrong targeting [7, 73]. This targeting strategy has never been tested in ovarian cancer.

### 7.2. Receptor-Specific Antibodies

These agents have been designed to be highly specific for the IGF-IR; that is, they do not bind to the insulin receptor. As described earlier, there exist hybrid receptors whose expression depends on the relative expression of the genes encoding the IGF-I and insulin receptors [73]. Based on this theory of “half receptors,” the novel antibody drug candidates have been designed to act against IGF-IR and hybrid receptors. Many have been studied in preclinical models and about a dozen are being evaluated in clinical trials simultaneously [7, 73, 74]. 

The first study targeting IGF-IR in ovarian cancer was published in 2003 by Hongo et al., in which they used a soluble form dominant negative of the type I IGF-IR designated 486/STOP in CaOV-3 cells [75]. This soluble IGF-IR is a truncated receptor at the 486th amino acid, located within the extramembranous *α*-subunit. They showed that the 486/STOP expression could reverse transformed phenotype of the CaOV-3 in vitro and inhibit tumorigenicity in vivo. Likewise, the administration of the 486/STOP recombinant protein retarded the tumor growth of CaOV-3 cells in vivo. 

Simultaneously, another group tested an antagonistic monoclonal antibody designated EM164, specific to the IGF-IR, in various cancer cell lines, including ovarian cancer [76]. They demonstrated a reduction of IGF-I-stimulated proliferation and survival of the human ovarian cancer OVCAR-5 cells.

### 7.3. Receptor Kinase Inhibitors

Small molecule inhibitors block IGF-IR activation by binding to the ATP-binding pocket of the receptor [77]. Most of the developed tyrosine kinase inhibitors have the side effect of attenuating insulin receptor signaling as well. However, despite this lack of specificity, they were found to be active in preclinical models and some are being evaluated in clinical trials [24, 74, 78]. There is a possibility that these agents might be more potent anticancer drugs since insulin receptor present on malignant cells may have an important role as well in carcinogenesis [7]. 

In the last couple of years, studies targeting IGF or insulin pathways in ovarian cancer mostly used small molecule IGF-IR kinase inhibitors. Indeed, our group reported an inhibition of cell survival in response to NVP-AEW541 in two human epithelial ovarian cancer cell lines, namely, OVCAR-3 and OVCAR-4 [38]. Interestingly, this effect was not reversible by the addition of recombinant IGF-I. We further demonstrated that this inhibitor sensitized cells to the effect of cisplatin, an effect described in other types of cancer cells as well [77]. This observation is relevant to the clinical application of the drug. Finally, NVP-AEW541 induced apoptosis and decreased AKT activation. We also performed a preliminary in vivo study using this small-molecule inhibitor in a human ovarian cancer xenograft model that gave promising results [79]. We confirmed our in vitro results using another IGF-IR kinase inhibitor produced by Bristol-Myers-Squibb, BMS-536924. BMS-554417 is a derivative of BMS-536924 and shares the same properties. Using the OV202 cells, Haluska et al. showed an antiproliferative effect of BMS-554417 at an IC50 of 7,5 *μ*M [80]. Moreover, the drug inhibited the phosphorylation of the IGF-IR, insulin receptor, AKT, and ERK1/2 and also induced apoptosis. In addition, treatment of OV202 with BMS-554417 stimulated the phosphorylation of HER-2. Inversely, treatment with the pan-HER inhibitor increased the phosphorylation of IGF-IR, suggesting a reciprocal cross-talk mechanism [81]. Therefore, the combination of BMS-536924 and a pan-HER inhibitor resulted in a synergistic antiproliferative effect in various ovarian cancer cell lines. A concomitant reduction of AKT and ERK phosphorylation and apoptosis induction were also demonstrated. Furthermore, HER receptor expression could confer resistance to IGF-IR-targeted therapy using breast cancer cells expressing HER-1 or HER-2. This suggests that combining targeted therapies to the HER and IGF-I family of receptors might be an effective strategy to overcome potential clinical resistance to IGF-IR inhibitors. 

Concurrently, we showed a dose and time-dependent growth inhibition of human epithelial ovarian cancer cell lines, the OVCAR-3 and OVCAR-4 in response to BMS-536924 [82]. This effect was partly mediated by AKT and the ribosomal protein S6. BMS-536924 provoked cell apoptosis as shown by the activation of PARP cleavage. We finally showed that this IGF-IR kinase inhibitor could sensitize cells to PARP inhibitors, possibly via the induction of DNA damage as indicated by the increased phosphorylation of histone H2AX. This study reinforced the concept that IGF-IR is a good therapeutic target in ovarian cancer. In addition, it proposes that combination therapy using BMS-536924 with a PARP inhibitor might be an effective strategy to circumvent resistance to treatment in clinical settings.

### 7.4. Metformin

Another potential drug targeting agent related to the insulin and/or IGF pathway is metformin. Metformin is an oral biguanide widely used since the 1950s for the treatment of type 2 diabetes, that lowers both circulating glucose and insulin levels. Two population studies provided preliminary evidence that metformin may reduce cancer risk and improve prognosis in patients with type 2 diabetes [83, 84]. Importantly, recent data demonstrated that the key mechanism of action of metformin is by activating the AMPK-LKB1 pathway [85, 86]. Other AMPK activators have been demonstrated to have growth inhibitory effects in various cancer cell types [87–89]. Therefore, metformin might have two potential antineoplastic effects: reducing circulating insulin levels and directly inhibiting growth through the AMPK-LKB1 pathway. 

We published the original study evaluating the antineoplastic effect of metformin in human epithelial ovarian cancer cell lines [90]. We demonstrated that metformin decreased in a dose and time-dependent manner ovarian cancer cells survival, an effect partly mediated by AMPK. Moreover, metformin potentiated the effect of cisplatin. The activation of AMPK by metformin was associated with an inhibition of downstream targets of AKT, such as phospho-p70S6 and phospho-S6. These findings led us to evaluate the potential applicability of metformin in the treatment of ovarian cancer by testing it in preclinical animal models. These experiments are currently underway of investigation in our laboratory. 

Only two other recent studies showed a cytotoxic effect of other AMPK activators. The first one is C93, a synthetic fatty acid synthase inhibitor that increased AMP/ATP ratio in SKOV3 human ovarian cancer cells, thereby provoking AMPK activation and leading to cell toxicity [91]. Using compound C, a specific inhibitor of AMPK, the authors clearly implicated AMPK in the cytotoxic action of C93. Interestingly, these findings were confirmed in vivo in an SKOV3 xenograft mice model [91]. The second study provided evidence that curcumin caused CaOV3 ovarian cancer cell death through AMPK, suggesting that the latter is a new molecular target of curcumin [92].

### 7.5. Clinical Trials

To the best of our knowledge, only two clinical trials using targeted therapy against IGF-IR are currently ongoing in ovarian cancer patients (clinicaltrials.gov identifier: NCT00719212 and NCT00718523). Both studies are testing the same human anti-IGF-IR human monoclonal antibody, namely, the AMG-479 [74, 93] that was previously tested clinically in other types of cancer [94, 95]. The objective of the first study is to verify whether the addition of AMG-479 to paclitaxel and carboplatin in first line chemotherapy could improve the progression-free survival in patients with optimally debulked FIGO stage III and IV ovarian epithelial carcinoma. The second study aims to obtain an estimate of the objective response rate (ORR) of AMG-479 in patients with recurrent platinum-sensitive ovarian epithelial carcinoma failing frontline chemotherapy. The completion dates of both studies are estimated in 2015 and 2012, respectively.

## 8. Conclusion

All members of the IGF family are expressed in malignant ovarian epithelial cells. On the other hand, circulating levels of IGF have not been undoubtedly associated with ovarian cancer risk or disease progression. However, a role of some of the components of the IGF family, such as IGF-I and IGF-IR, has been clearly involved in ovarian tumorigenesis. In the past few years, various inhibitors of IGF-IR have been developed, including AMPK activators. These were tested in ovarian cancer in vitro and in vivo models, obtaining promising results for the potential of this targeted strategy in ovarian carcinoma, supported by the currently ongoing clinical trials.

## Figures and Tables

**Table 1:** Normal physiological role of insulin/IGF family members.

	Origin	Gene regulation	Serum levels	Affinity for insulin/IGF family members	Function
Insulin	Beta cells of the pancreas	Glucose uptake, protein synthesis	11–14 *μ*U/mL	IR >IGF-IR> IGF-IIR	–Regulates carbohydrate, fat, and protein metabolism.
					–Mitogenic effect.

IGF-I	Liver, bone, and several other tissues.	Hormones, growth factors, cytokines, nutrition, smoking, exercise.	100–200 ng/mL	IGF-IR> IGF-IIR > IR	–Regulates embryonic growth and specific differentiation in adult tissues.
					–Involved in cell proliferation, transformation, and antiapoptotic activity.

IGF-II	Liver, kidney, bone, and several other tissues.	Tumor suppressor proteins WT1 and p53, HIF-1, genomic imprinting.	400–700 ng/mL	IGF-IIR> IGF-IR>IR	–Functional during embryonic and fetal growth.

IGFBPs	IGFBP-1: liver, decidua.	BP-1: insulin, steroids.	BP-1, BP-2: vary during the day and meals.	BP-1, BP-3, BP-5: IGF-I> IGF-II	–Regulate transport and half-life of IGFs between different body compartments.
	IGFBP-2: CNS.	BP-2: insulin, metabolic process.			
	IGFBP-3: several tissues.	BP-3: GH, PTH, cytokines, p53, estradiol, steroids.			
	IGFBP-4: bone, CNS, prostate.	BP-4: vitamin D, parathyroid hormone.	BP-3: vary in relation to age and sex 1500–5580 ng/mL.	BP-2, BP-6: IGF-II>IGF-I	–Regulate IGF independent effect on cell proliferation and apoptosis.
	IGFBP-5: kidney, bone, mammary gland.	BP-5: GH, prolactin, vitamin D.			
	IGFBP-6: ovary, prostate.	BP-6: GH, FSH.			

**Table 2:** Tissue expression modulations of the insulin/IGF system in ovarian cancer.

Insulin/IGF components	No. of patients	Modulation	Reference
Free IGF-I mRNA and protein	215 EOC	↑	[43]
IGF-I, IGF-IR mRNA, and several genes downstream of the receptor	64 EOC	↑	[44]
IGF-II mRNA	109 EOC	↑	[45]
IGF-II mRNA	215 EOC	↑	[46]
IGFBP3 protein	128 clear cell carcinoma	↑	[47]
IGFBP-2 mRNA	113 EOC	↑	[48]
IGFBP-3 protein	147 EOC	↓	[50]
IGFBP-3 protein	35 endometrioid carcinoma	↓	[51]

EOC: epithelial ovarian cancer.

**Table 3:** Circulating protein levels of the insulin/IGF system in ovarian cancer.

Insulin/IGF components	No. of patients	Modulation	Reference
IGFBP-2	20 EOC	↑	[49]
IGF-I	58 EOC	↓	[53]
IGF-I	24 EOC	↓	[52]
IGF-I	59 EOC	↓	[54]
IGF-I	9 EOC	↓	[55]
IGF-I	132 EOC (<55 yrs.)	↑	[56]
IGF-I	214 EOC (<55 yrs.)	↑	[57]
IGF-I, IGFBP-2, IGFBP-3	222 EOC	↔	[58]

EOC: epithelial ovarian cancer.
